# Unilateral T-shaped incomplete duplex nephrectomy in an adolescent under laparoscopy: A case report

**DOI:** 10.1097/MD.0000000000025187

**Published:** 2021-03-26

**Authors:** HuaJie He, YuanBi Huang, Lu Yu, QiGuang Li, Xian Long, YongPeng Li, RongChao Chen, XianLin Yi

**Affiliations:** a Department of Urology, Cancer Hospital of Guangxi Medical University & Guangxi Cancer Research Institute, Nanning; b School of Nursing, Kunming Medical University, Kunming, China.

**Keywords:** adolescent, laparoscopic surgery, repeated kidney

## Abstract

**Introduction:**

Duplicate kidneys are the most common congenital abnormality of the urinary system. The location of duplicate kidneys varies. We report a case of an adolescent with upper and lower kidneys that are arranged vertically and approximately T-shaped.

**Patient concerns:**

A 16-year-old male teenager was examined for pain in the left side of the waist. The Computed Tomography scan revealed that the left kidney was incompletely duplicated and fused; the left upper urinary tract was incompletely obstructed.

**Diagnosis:**

The abdominal tomography confirmed the diagnosis of incomplete duplicate kidney.

**Interventions:**

The patient underwent laparoscopic surgery. The failure to ligate the renal pedicle resulted in increased bleeding during the operation and an open ureteral stump.

**Outcomes:**

No urine leakage occurred after the operation. Doppler ultrasound of the urinary system showed no hydronephrosis, and the patient was asymptomatic.

**Conclusion:**

Through this case report, we found that the duplicate kidneys could be arranged in a T-shape under laparoscopy. Although only the supply of the duplicate renal arteries can be ligated during surgical resection, the renal pedicle must also be ligated during the operation if there is a lot of bleeding.

## 1 Introduction

The incidence of duplicate renal ureteral malformations is about 0.8%. Duplicate renal ureteral malformations are commonly reported in children and rare in adults and generally divided into complete and incomplete.^[1]^ Congenital renal abnormalities are rare and generally have no evident symptoms, and most of these abnormalities are incidentally found with other diseases. Renal malformations and duplications have no specific manifestation and are often complicated by other urinary system malformations, such as ureteral orifice cysts, obstruction of the renal pelvic and ureteral junctions, ureteral stenosis, vesicoureteral reflux and other malformations.^[2,3]^ Renal malformations and duplication easily cause obstructive hydronephroureter, infection, stone formation and related symptoms and signs. Duplicate kidneys can be separate from normal kidneys or partially fused.^[4]^ In most cases both the kidneys are located on the same side and in a fused state. Two separate ureters are present in each kidney. In complete duplication of kidney and ureter, the normal and the abnormal ureters open in the bladder and other parts, respectively. The ureteral opening of the upper kidney is generally lower than that of the lower kidney. The hemi-nephrectomy is an effective treatment method, wherein the non-functional part of the kidney is removed from the duplicate kidney.^[3]^ The key point of the nephrectomy of the duplicate kidney is to fully expose the renal pedicle vessels during the operation.^[6,7]^

However, current case reports are based on imaging, and the intuitiveness under laparoscopy is lacking. We report an adolescent male with a T-shaped incomplete duplicate kidney, which is partially fused and has a separate ureter, who received laparoscopic treatment.

## 2 Case report

**Figure 1:**
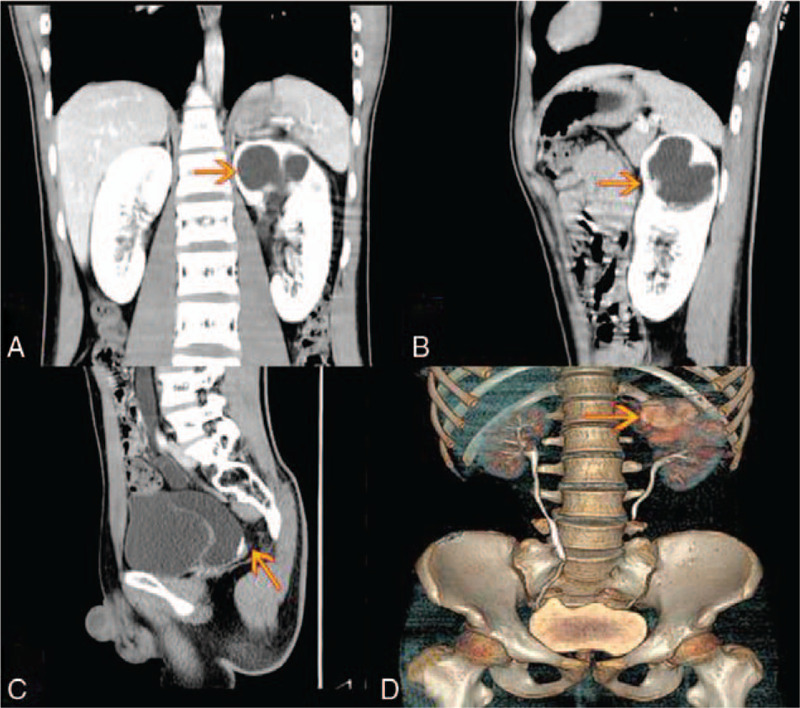
A 16-year-old male teenager with duplicate kidneys. A, Coronal B, Left sagittal contrast-enhanced CT C, Left ureteral lower calculi D, CTU showed the left ureteral duplication malformation (arrow).

A 16-year-old adolescent male with low back pain was diagnosed with left hydronephrosis and left lower ureteral calculi by abdominal ultrasound examination. Abnormal elevation of the uric acid was observed in the laboratory examination, and no stone was observed on ureteroscopy. A ureteral stent was placed. The computed tomography (CT) of the abdomen showed 2 sets of renal pelvis and ureter in the left kidney (Fig. 1A). The upper kidney was smaller, and the lower kidney was contained hydrops. The included angle of the upper and lower kidneys was obtuse (Fig. 1b). The ureteral pelvic segment (after confluence) was observed with a stone shadow with size of 0.8 cm × 0.3 cm (Fig. 1C). The CT urography showed left ureteral duplication malformation (Fig. 1D). We finally diagnosed that this patient had an incomplete duplication renal malformation. The renal emission CT revealed that the blood perfusion in the upper middle and the upper left kidneys was decreased, and the secretion function of the left kidney was normal. The filtration function was slightly impaired. Water accumulation was observed in the middle and the upper kidneys and the left ureter. Incomplete obstruction of the left upper urinary tract was observed.

**Figure 2:**
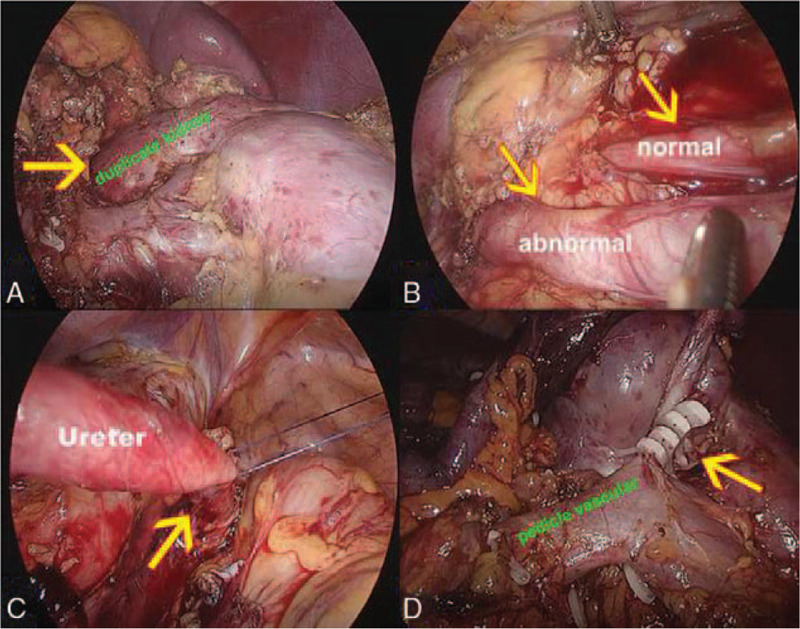
Laparoscopic vision: A, duplicate kidneys is similar to T-shape and partially fused (arrow) B, Normal and abnormal ureters (arrow) C, abnormal ureters opened around the bladder and the prostate (arrow) D, Block branch vessel (arrow)and removal of duplicate kidney.

**Figure 3:**
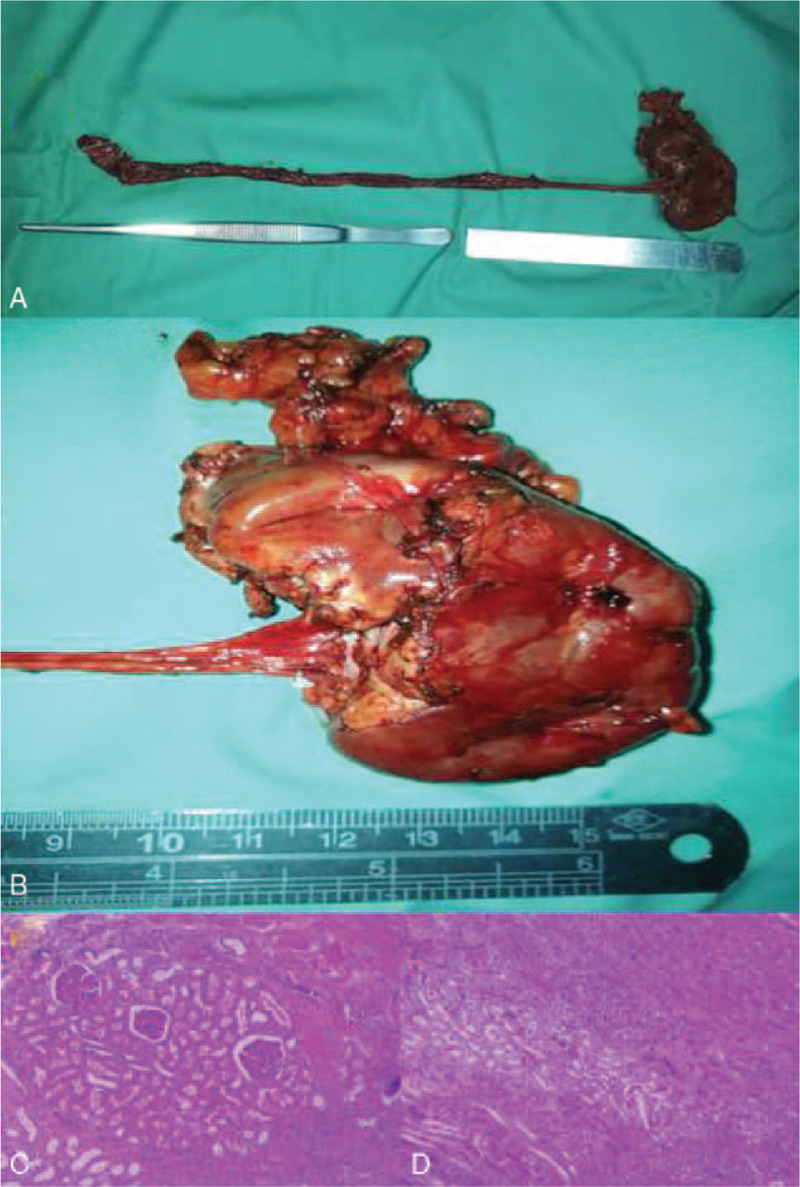
The specimen: A, duplicate kidney and ureter B, Kidney with intact capsule C and D, cystic dilation and atrophy, reduction of nephrons, glomerular capillary atrophy, and reduced number of renal tubules.

We decided to perform laparoscopic surgical resection of the upper kidney and the ureter. During the operation, the upper and the lower kidneys were vertically arranged in a similar T-shape and partially fused (Fig. 2A). The upper kidney had an independent ureter (Fig. 2B) and opened around the bladder and the prostate where it tortuously expanded (Fig. 2C). The ureter was excised below the iliac blood vessel, and the stump was kept open. The stone was removed, and the hilar blood vessel was freed. The secondary branch was separated and clipped. The upper half of the kidney was removed (Fig. 2D), and the bleeding was about 500 ml. The wound was sutured continuously with barbed thread to stop the bleeding, and the operation field was closed after the specimen was collected (Fig. 3A, B). The postoperative procedures were uneventful, and the drainage fluid was less than 10 ml. The drainage tube was removed four days after the operation. No leakage of urine was observed. The wound healing was delayed due to infection. The histological diagnosis was consistent with the duplicate renal malformation, which manifested as cystic dilation and atrophy, reduced number of nephrons, glomerular capillary atrophy, and reduced number of renal tubules under the microscope (Fig. 3C, D). The CT scan after 10 days showed the left independent collection system. At 1 month follow-up, the coloured Doppler ultrasound of the urinary system showed no hydronephrosis, and the patient was asymptomatic.

## 3 Discussion

Duplicate kidneys are the most common congenital abnormality of the urinary system and reported mostly in children. Duplicate kidneys are generally discovered incidentally. The embryological basis of duplicate kidneys is the abnormal division of the nephrogenic cord into 2 metanephric blastocysts, thereby forming 2 kidneys.^[8]^ The partial or the complete duplication of ureteral buds is involved and can be separated from the normal kidney or partially fused.^[4]^ Bilateral kidneys are mostly on the same side and fused in most cases. Two separate ureters originate from their respective kidneys.^[5]^ In this case, the upper and the lower kidneys were perpendicular to each other and arranged in an approximate T-shape.

Hemi-nephrectomy is an effective treatment method. The nonfunctional part of the kidney is removed from the duplicate kidney.^[3]^ In patients with complete duplicate renal ureteral malformations, the normal and the abnormal ureters open in the bladder and other parts, respectively, and the ureter opening of the upper kidney is generally lower than that of the lower kidney.^[1]^ The dysplasia of the upper half is more common than that of the lower half. Bilateral dysplasia is rare in the kidneys. The upper pole is susceptible to abnormal ureter formation, including ectopic and ureteral cysts.^[9]^ In this case report, the abnormal and the normal ureters were independent and tortuously dilated. The abnormal ureter opened in the seminal vesicle area of the bladder and was accompanied by the stone formation. The ureter opened in the triangle of the bladder seminal vesicle. The upper kidney showed shrinkage and expansion with water.

The renal blood supply of the duplicate kidney often has the following conditions^[10]^

(1) The main trunk is in the lower renal blood vessels, which branch out to the upper kidney.
(2) A main trunk originates from the abdominal aorta and the vena cava and branches up and down to the upper and the lower kidneys, respectively.
(3) The upper and the lower renal blood vessels arise directly from the abdominal aorta or the vena cava.

In this patient, the blood vessel originated from the abdominal aorta and the vena cava and branched up and down to the upper and the lower kidneys. The most important thing was to keep the upper ureteral wall open as much as possible to keep the blood flowing to the remaining lower ureter.^[9]^ The standard hemi-nephrectomy involves separating the blood vessels at the hilus of the kidney and ligating the blood vessels that supply the upper kidney. A successful hemi-nephrectomy depends on the maximum protection of the function of the lower kidney.^[7]^ No unified opinion exists on whether ligating the renal pedicle is necessary and hence it should be decided in accordance with the intraoperative situation.

We did not ligate the renal pedicle during the operation, and hence increased bleeding was observed. Ligation might cause damage to the function of the remaining half of the kidney, which might cause short- or long-term surgical complications. Our experience taught us that we should be prepared to ligate the renal pedicle during the operation. For upper nephrectomy of the duplicate kidney, the upper renal pelvis should be removed as completely as possible. When the connected renal parenchyma is excised, it should be done slightly close to the upper renal parenchyma to prevent the loss of normal lower nephrons. The postoperative leakage of urine and urinary cysts are often caused by renal pelvic mucosal tissue residue of the duplicate kidney.^[11]^ Therefore, the renal pelvic mucosa must be completely removed or destroyed by electrocautery to avoid postoperative leakage of urine.

If the lower renal ureter has no evident function and has a normal shape, the ureteral collateral anastomosis can be performed.^[11]^ The anastomotic leakage after operation is one of the serious complications of ureteral bladder replantation.^[7]^ In this case, the ureter of the duplicate kidney opened in the triangle of the seminal vesicle of the bladder. No ureteral stump syndrome was observed after the operation. In this case, the drainage fluid < 10 ml/day was acceptable.

Complications of nephrectomy of the duplicate kidney include bleeding, leakage of urine, allantoic cyst, remaining renal function damage or even renal function loss.^[9]^ Postoperatively, patients develop fever and abdominal wound infection, leading to delayed healing. The main reason for this can be intra-operative leakage of urine into the abdominal cavity when the renal pelvis is removed.

## Acknowledgments

No support from any organisation for the submitted work; no financial relationships.

## Author contributions

HuaJie He, YuanBi Huang, are joint first authors. Study design: HuaJie He YuanBi Huang Xianlin Yi. Purchase experimental materials, record and organize data: Lu Yu, QiGuang Li, Xian Long, YongPeng Li, RongChao Chen, Drafting of the manuscript: HuaJie He Xianlin Yi. All authors have read and approved the final manuscript.

**Conceptualization:** HuaJie He, YuanBi Huang, Rongchao Chen.

**Data curation:** QiGuang Li, Xian Long, Yongpeng Li.

**Formal analysis:** HuaJie He, Lu Yu, Rongchao Chen.

**Funding acquisition:** Xian Long, XianLin Yi.

**Investigation:** HuaJie He, YuanBi Huang, Lu Yu, Rongchao Chen.

**Methodology:** HuaJie He, YuanBi Huang, QiGuang Li, Xian Long, Yongpeng Li, Rongchao Chen.

**Project administration:** HuaJie He, QiGuang Li, Yongpeng Li.

**Resources:** YuanBi Huang, Xian Long, Yongpeng Li, Rongchao Chen, XianLin Yi.

**Software:** HuaJie He, Yongpeng Li.

**Supervision:** XianLin Yi.

**Visualization:** XianLin Yi.

**Writing – original draft:** HuaJie He, YuanBi Huang, Lu Yu.

**Writing – review & editing:** Yi XianL in.
